# Differences in clinical characteristics and cardiovascular disease risk prediction among Chinese women with polycystic ovary syndrome phenotypes: a cross-sectional study

**DOI:** 10.3389/fendo.2026.1753316

**Published:** 2026-04-10

**Authors:** Ziye Gong, Danyang Li, Xuan Li, Jinjin Tian, Jing Guo, Yuying Zhao, Yin Lu, Shan Gao, Ming Li

**Affiliations:** 1 Department of Endocrinology, Xuanwu Hospital Capital Medical University, Beijing, China; 2 Department of Endocrinology, National Health Commission (NHC) Key Laboratory of Endocrinology, Peking Union Medical College Hospital, Peking Union Medical College and Chinese Academy of Medical Sciences, Beijing, China

**Keywords:** cardiovascular disease risk, China-PAR model, metabolic abnormalities, obesity, phenotypes, polycystic ovary syndrome

## Abstract

**Background:**

Polycystic ovary syndrome (PCOS) is associated with increased cardiovascular disease (CVD) risk, but differences across phenotypes in Chinese women remain unclear. This study aimed to characterize clinical profiles of PCOS phenotypes, predict CVD risks, and evaluate associations between phenotypes and CVD risk.

**Methods:**

A total of 206 women with PCOS were included from an initial cohort of 211 and classified into four phenotypes according to Rotterdam criteria. Clinical data, laboratory results, and imaging measurements were collected. CVD risks were estimated using the China-PAR model. One-way ANOVA and the Kruskal-Wallis test were used for continuous variables, and Pearson’s chi-square or Fisher’s exact test for categorical variables. Firth logistic regression was employed to assess the association between PCOS phenotypes and CVD risk, and mediation analysis detected the indirect effects.

**Results:**

Among 206 patients with PCOS, 104 (50.5%), 36 (17.5%), 19 (9.2%) and 47 (22.8%) were classified as phenotype A, B, C and D. BMI, WC, SBP, and DBP were significantly higher in phenotypes A, B, and C than in D (P<0.05). UA, LDL-C, TG, and HOMA-IR were significantly higher in phenotypes A and B than in D, while HDL-C and ISI-Matsuda were significantly lower (P<0.05). Lifetime CVD risk scores were significantly higher in phenotypes A, B, and C compared with D (P< 0.05), with values of 15.55%, 17.65%, 17.30%, and 9.90%. After adjusting for diet, physical activity, and medication use, phenotypes A (OR 3.18, 95% CI: 1.30-8.84, P = 0.010), B (OR 4.90, 95% CI: 1.58-16.44, P = 0.006), and C (OR 4.67, 95% CI: 1.39-16.64, P = 0.013) were significantly associated with higher odds of high lifetime CVD risk compared with D. The mediating effects of BMI, HOMA-IR, and UA were significant (P< 0.05), with BMI exhibiting the largest mediating effect, accounting for 94.7%, 70.9%, and 42.9% of the total effect in phenotypes A, B, and C compared with D.

**Conclusions:**

Our results demonstrated that more adverse clinical abnormalities and significantly higher CVD risk in women with phenotypes A, B, and C compared with D. BMI, HOMA-IR, and UA play a significantly mediating role between phenotypes and CVD risk. This study suggests that it may be necessary to conduct regular CVD risk assessments for patients with different phenotypes of PCOS, in order to guide early individualized treatment strategies, with a focus on weight and metabolic management.

## 1 Introduction

PCOS is the most common endocrinopathy in reproductive-aged women, with impacts throughout the lifespan, from adolescence to postmenopause. Its estimated prevalence ranges from 10% to 13% (1). The primary clinical features of PCOS include menstrual irregularities such as oligomenorrhea and amenorrhea, as well as hyperandrogenism (HA), which may present as hirsutism, hair thinning, or acne vulgaris (2).

In addition to the classic reproductive manifestations, most women with PCOS also exhibit metabolic abnormalities, such as insulin resistance (IR), overweight and obesity, and dyslipidemia, which significantly elevate the risk of metabolic diseases and further increase the risk of cardiovascular disease (CVD) (3, 4). For instance, a Danish registry cohort study reported a 19% increased risk of incident CVD in women with PCOS (5). Similarly, a meta-analysis revealed that women with PCOS had a 1.3-fold higher risk of developing composite CVD compared to women without PCOS (6).

Accurate prediction of individual CVD risk is essential for the prevention and management of this significant health condition. However, only a limited number of studies have applied predictive models to estimate CVD risk specifically in women with PCOS. Amiri et al. (7) used the Framingham Risk Score (FRS) in Iranian women with PCOS and reported a significant increase in CVD risk. Similarly, Zeng et al. (8) applied the China-PAR risk model to Chinese women with PCOS and found that late-night habits further elevated CVD risk. Additionally, several studies have suggested that CVD risk is not uniform across all women with PCOS, with higher risk observed in certain subtypes—particularly the classic phenotype, characterized by menstrual irregularities and HA—especially when compounded by obesity and diabetes (9, 10).

Currently, we have not found any studies that systematically compare CVD risk across the four PCOS phenotypes (A, B, C, and D) in Chinese women, nor any studies that apply predictive models to estimate CVD risk within these subgroups. Therefore, this study aimed to characterize the clinical profiles of different PCOS phenotypes, apply the China-PAR risk model to estimate CVD risks across phenotypic subgroups, and explore the association between PCOS phenotypes and CVD risk.

## 2 Materials and methods

### 2.1 Subjects

**Figure 1:**
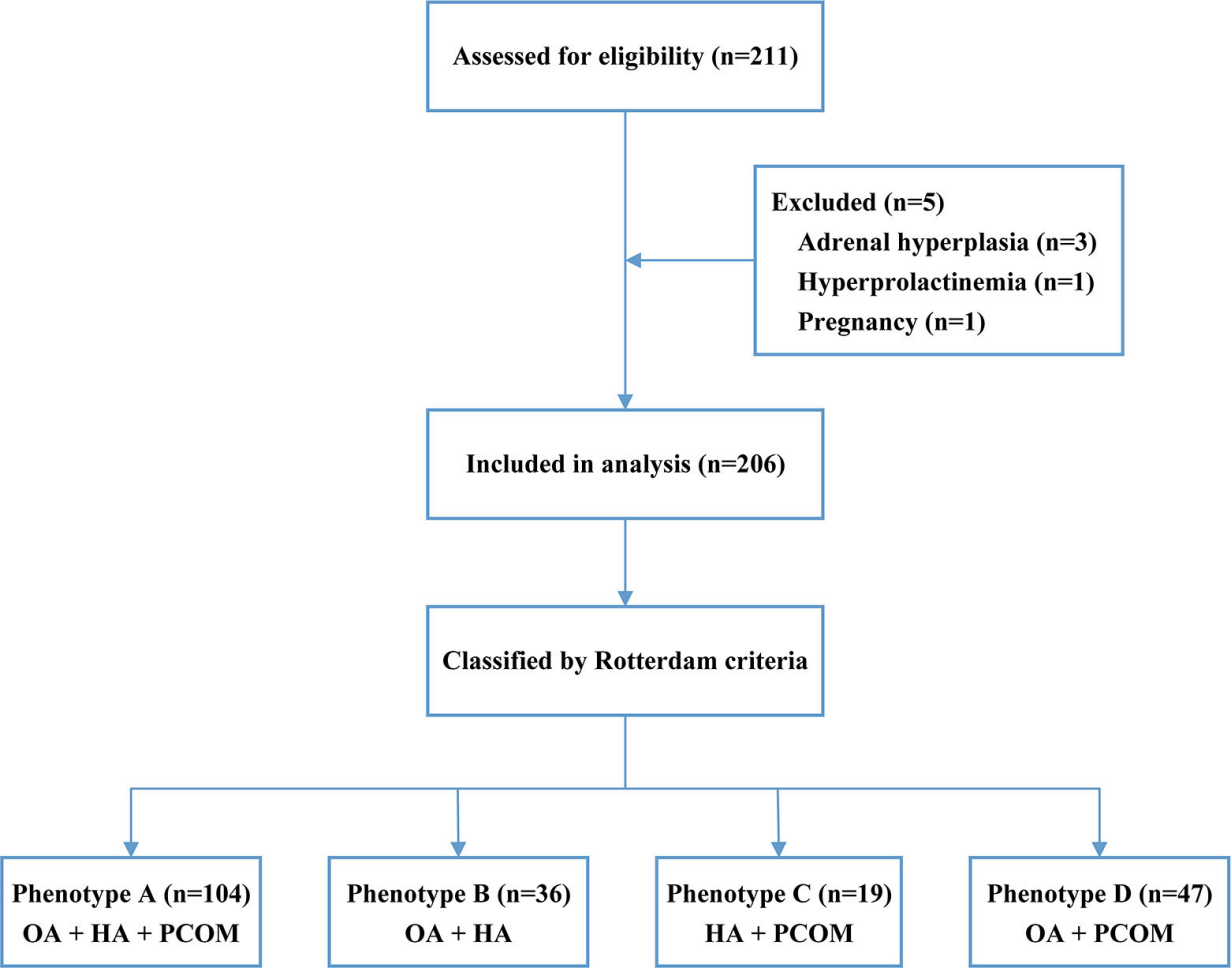
Flow of participants through the study. A total of 211 women with PCOS were assessed for eligibility, of whom five were excluded (three with adrenal hyperplasia, one with hyperprolactinemia, and one due to pregnancy). Consequently, 206 women were included in the analysis, and were classified into the following Rotterdam phenotypes: phenotype A (n = 104), phenotype B (n = 36), phenotype C (n = 19), and phenotype D (n = 47).

A total of 211 women aged 20–50 years, diagnosed with PCOS according to the Rotterdam criteria, were recruited from the Department of Endocrinology, Xuanwu Hospital, Capital Medical University, between June 2024 and July 2025 (**Figure 1**). The Rotterdam diagnostic criteria require the presence of at least two of the following three features: HA (clinical or biochemical), oligo/anovulation (OA), and polycystic ovarian morphology (PCOM) (11). PCOM is defined by ultrasound as the presence of ≥12 follicles measuring 2–9 mm per ovary or an ovarian volume ≥10 mL (11), and was assessed by a single experienced ultrasound physician to ensure consistency and reliability.Based on these criteria, PCOS is classified into four phenotypes: phenotype A (OA + HA + PCOM), phenotype B (OA + HA), phenotype C (HA + PCOM), and phenotype D (OA + PCOM) (12). Exclusion criteria included hyperandrogenism due to other causes (e.g., Cushing’s syndrome, non-classic congenital adrenal hyperplasia, or androgen-secreting tumors), ovulatory dysfunction of non-PCOS origin (e.g., functional hypothalamic amenorrhea, hyperprolactinemia, or premature ovarian insufficiency), current pregnancy or lactation, and regular use of oral contraceptives, metformin, or glucagon-like peptide-1 (GLP-1) receptor agonists in the past 3 months.

### 2.2 Clinical data collection

Clinical data were collected by a single experienced researcher to ensure consistency and reliability. Information obtained included sociodemographic characteristics (age and residential area), clinical manifestations (menstrual disorders; hirsutism, defined as a Ferriman–Gallwey score ≥ 5 (13); acne vulgaris), current smoking status, medical history (antihypertensives and lipid-lowering drugs), family history of CVD, anthropometric measurements dietary and physical activity information. Anthropometric assessments were conducted as follows: height (cm) was measured using a stadiometer; weight (kg) was obtained with a mechanical scale; and waist circumference (WC, cm) was measured horizontally 1 cm above the umbilicus with a non-elastic tape, with central obesity defined as WC ≥ 85 cm (14). Systolic and diastolic blood pressure (SBP and DBP) were measured using a validated, calibrated mercury sphygmomanometer after participants had refrained from exercise for at least 30 minutes, with hypertension defined as BP ≥ 140/90 mmHg (15). All measurements were taken in duplicate, and mean values were used for analysis. Body mass index (BMI) was calculated as weight in kilograms divided by height in meters squared (kg/m²), with overweight defined as BMI ≥ 24.0 kg/m² and obesity defined as BMI ≥ 28.0 kg/m² (14). Dietary intake was assessed using a semi-quantitative food frequency questionnaire (FFQ) focusing on the frequency of fried foods, sweets, and sweetened drinks (16). Scoring for the frequency categories was performed, and participants were categorized into a combined low-moderate group and a high group based on tertiles, with higher scores indicating a less healthy diet characterized by higher sugar and fat intake. Physical activity was assessed using the International Physical Activity Questionnaire Short Form (IPAQ-SF) (17), where the total physical activity score was calculated by multiplying the duration and frequency of each activity, then converting into Metabolic Equivalent of Task (MET)-minutes per week using standard MET values (vigorous: 8, moderate: 4, walking: 3.3). Participants were categorized into a low activity group and a combined moderate-high activity group based on their total MET-minutes per week and activity type.

### 2.3 Laboratory and imaging measurements

Following a 12-hour overnight fast, serum samples were obtained from all participants. A 75g oral glucose tolerance test (OGTT) was performed to assess glucose metabolism and insulin resistance (IR), with blood samples collected at 0 and 2 hours for glucose and insulin measurements. Biochemical parameters including fasting blood glucose (FBG, mmol/L), 2-hour blood glucose (2-h BG, mmol/L), uric acid (UA,μmol/L), triglycerides (TG, mmol/L), total cholesterol (TC, mmol/L), high-density lipoprotein cholesterol (HDL-C, mmol/L), and low-density lipoprotein cholesterol (LDL-C, mmol/L) were analyzed using an automatic biochemical analyzer (LABOSPECT 008 α, Hitachi Ltd., Japan). Fasting insulin (FINS, μIU/mL) and 2-hour insulin (2-h INS, μIU/mL) were measured by electrochemiluminescence immunoassay (Cobas e601, Roche Inc., USA). Glycated hemoglobin (HbA1c, %) was quantified via high-performance liquid chromatography (VARIANT II Turbo, Bio-Rad Inc., USA). For women with regular menstrual cycles, sex hormone measurements were performed on days 2–3 of the menstrual cycle. Follicle-stimulating hormone (FSH, mIU/mL), luteinizing hormone (LH, mIU/mL), total testosterone (TT, ng/dL) and anti-Müllerian hormone (AMH, ng/mL) were assessed using chemiluminescence immunoassay (DxI800, Beckman Coulter Inc., USA). Ovarian morphology and follicle count were evaluated by transvaginal or abdominal ultrasound (LOGIQ E9, GE HealthCare, USA).

IR was assessed using the homeostatic model assessment of IR (HOMA-IR). HOMA-IR was calculated using the following formula (18):


$$\frac{Fasting\;insulin\;\left(\mu U/mL\right)\times Fasting\;glu\cos e\;\left(mmol/L\right)}{22.5}$$

HOMA-IR >2.69 was defined as insulin resistance (19).

Insulin sensitivity was assessed using the Matsuda index (ISI-Matsuda). ISI-Matsuda was calculated using the following formula (20):


$$\frac{10,000}{\sqrt{\text{Fasting glucose }\left(\text{mg}/\text{dl}\right)\times \text{Fasting insulin }\left(\text{μU}/\text{ml}\right)\times \text{Mean OGTT glucose}\times \text{Mean OGTT insulin}}}$$

Impaired glucose metabolism, including prediabetes and diabetes mellitus, was diagnosed according to the 2025 American Diabetes Association Standards of Medical Care in Diabetes (21). Hyperuricemia was defined as a serum UA level >360μmol/L (22). Dyslipidemia was diagnosed if one or more of the following lipid abnormalities were present: TC ≥5.2 mmol/L, TG ≥1.7 mmol/L, HDL-C<1.3 mmol/L, or LDL-C ≥3.4 mmol/L (23). Metabolic syndrome (MetS) was defined as the presence of at least three of the following: central obesity; FPG≥5.6 mmol/L; blood pressure ≥130/85 mmHg; TG ≥1.70 mmol/L; or HDL-C<1.3 mmol/L (24, 25).Hyperandrogenemia was defined as a TT level >75 ng/dL.

### 2.4 Cardiovascular risk assessment

Cardiovascular risk was evaluated using the China-PAR risk model (26), a validated algorithm specifically developed for Chinese populations to estimate both 10-year and lifetime risks of CVD. This model incorporates multiple risk factors, including age, sex, residential area, blood pressure, WC, HDL-C, TC, current smoking status, diabetes mellitus, family history of CVD and the use of antihypertensive drugs. Based on model outputs, participants were stratified into risk categories as follows (1): 10-year CVD risk: high (≥10.0%), intermediate (5.0–9.9%), or low (<5.0%); and (2) lifetime CVD risk: high risk (top tertile) or low risk (remaining two tertiles) (8, 27).

### 2.5 Statistical analyses

SPSS version 20.0 and GraphPad Prism 9.0 were used for data analysis and graph construction. Normality was assessed using the Shapiro-Wilk test, along with visual inspection of P-P and Q-Q plots. Normally distributed continuous variables were presented as mean ± SD, non-normally distributed variables were expressed as median (25th percentile, 75th percentile). One-way ANOVA was used for normally distributed continuous variables, with *post-hoc* pairwise comparisons using the LSD method. For non-normally distributed continuous variables, the Kruskal-Wallis test was applied, with Bonferroni correction for multiple comparisons. Categorical variables were analyzed using Pearson’s chi-square or Fisher’s exact test, with *post-hoc* pairwise comparisons adjusted by Bonferroni correction. Spearman’s rank correlation analysis was used to examine the correlations between lifetime CVD risk score and clinical parameters.Since age, blood pressure, WC, HDL-C and TC are components of the China-PAR formulation, they are not included as confounding factors in further analyses. R version 4.5.2 with the logistf and mediation packages was used to perform Firth logistic regression and mediation analysis. Given the relatively small sample size in the study, Firth logistic regression was used to examine the association between PCOS phenotypes and high lifetime CVD risk, with odds ratios (OR) and 95% confidence intervals (CI) calculated. Diet, physical activity, and medication use, recognized as factors influencing CVD risk, were adjusted for as potential confounders (28). Mediation analysis was performed to evaluate the indirect effects of metabolic factors on the association between PCOS phenotypes and high lifetime cardiovascular disease risk, employing bootstrap resampling.

## 3 Results

### 3.1 Clinical characteristics in women with different PCOS phenotypes

A total of 206 women with PCOS were included in the study, with 5 excluded. Among them, 104 (50.5%), 36 (17.5%), 19 (9.2%), and 47 (22.8%) were classified as phenotypes A, B, C, and D (**Figure 1**). The distribution of clinical manifestations varied significantly among the four phenotypes. Menstrual disorders were present in phenotypes A, B, and C, while phenotype D had no cases of menstrual disorders (P<0.001). Hirsutism was most common in phenotype B (36, 100%), followed by A (100, 96.2%), C (16, 84.2%), and not observed in D (P<0.001). Acne vulgaris occurred most frequently in phenotype A (72, 69.2%), followed by C (11, 57.9%), B (20, 55.6%), and D (14, 29.8%) (A vs D, P<0.001).

**Figure 2:**
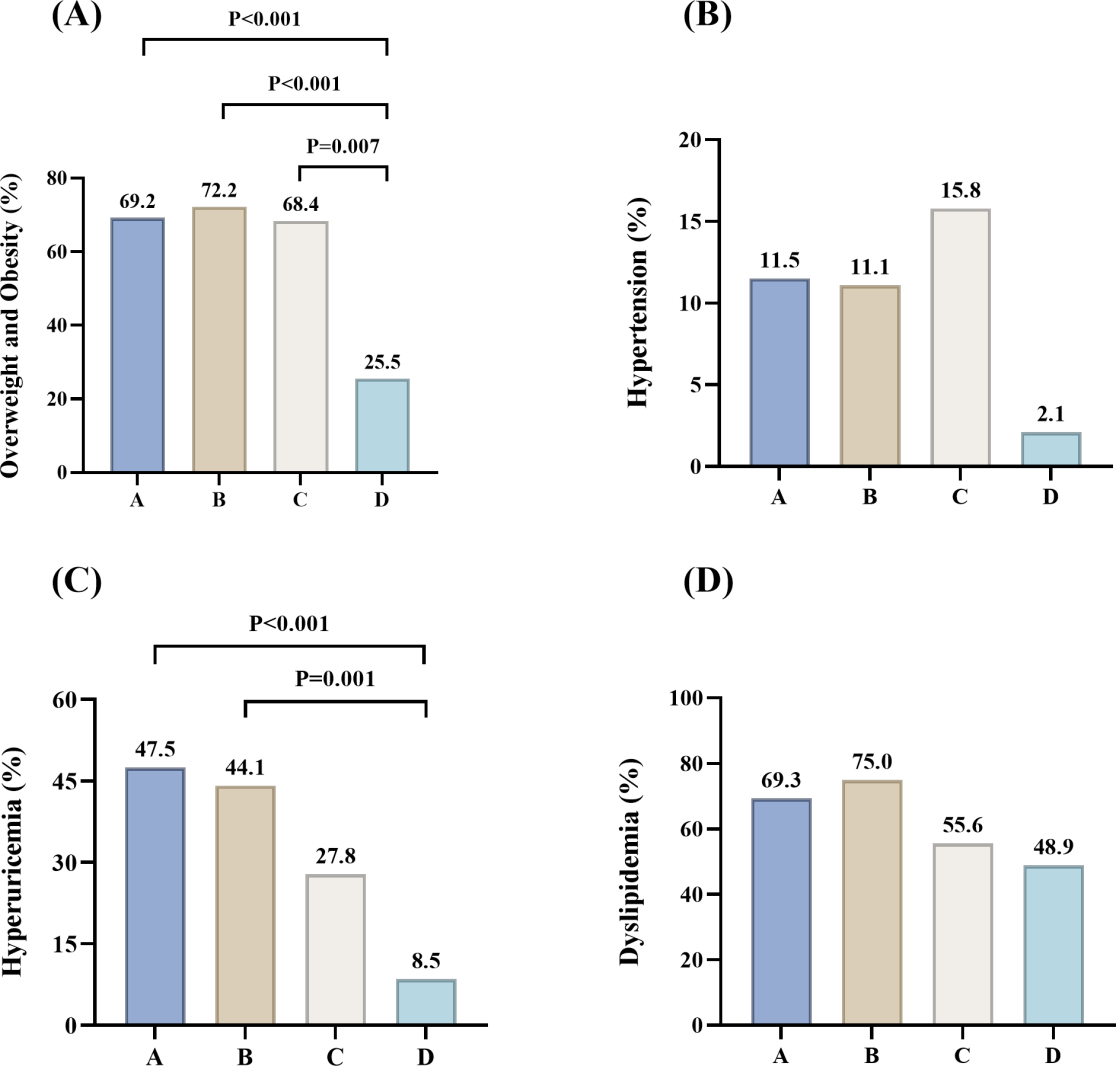
Proportions of women with overweight and obesity, hypertension, hyperuricemia, and dyslipidemia across different PCOS phenotypes. **(A)** Overweight and obesity; **(B)** Hypertension; **(C)** Hyperuricemia, with 200 patients, including 101, 34, 18, and 47 in phenotypes A, B, C, and D; **(D)** Dyslipidemia, with 198 patients, including 101, 32, 18, and 47 in phenotypes A, B, C, and D. P< 0.05 was considered significant.

As presented in **Table 1**, significant differences in clinical characteristics were observed across the PCOS phenotypes. Women with phenotypes A, B, and C had significantly higher BMI and WC compared with those with phenotype D (BMI: P<0.001, P<0.001, P = 0.020; WC: P<0.001, P<0.001, P = 0.026). The prevalence of overweight and obesity was higher in phenotype B (26, 72.2%), A (72, 69.2%) and C (13, 68.4%) compared with D (12, 25.5%) (P<0.001, P<0.001 and P = 0.007) (**Figure 2A**). SBP and DBP were significantly higher in phenotypes A, B, and C compared with D (SBP: P< 0.001, P<0.001, P = 0.006; DBP: P = 0.002, P = 0.008, P = 0.012). The prevalence of hypertension was highest in phenotype C (3, 15.8%), followed by A (12, 11.5%), B (4, 11.1%), and D (1, 2.1%), with no statistically significant differences observed (**Figure 2B**). UA levels were significantly higher in phenotype B compared with C and D (P = 0.048 and p<0.001). Phenotype A also had significantly higher UA levels than D (p<0.001).The prevalence of hyperuricemia was highest in phenotype A (48, 47.5%), followed by B (15, 44.1%), C (5, 27.8%), and D (4, 8.5%) (A vs D, P<0.001; B vs D, P = 0.001) (**Figure 2C**). HDL-C levels were significantly lower in phenotypes A and B compared with D (both P<0.001). LDL-C levels were significantly higher in phenotype B than in A, C, and D (P = 0.030, P = 0.036, and P< 0.001). Phenotype A also had significantly higher LDL-C levels than D (P = 0.037). In addition, TG levels were significantly higher in phenotypes A and B than D (P = 0.019 and P = 0.009). Phenotype B exhibited the highest proportion of dyslipidemia (25, 75.0%), followed by A (70, 69.3%), C (10, 55.6%), and D (23, 48.9%), with no statistically significant differences observed (**Figure 2D**).

**Table 1:** Clinical characteristics and CVD risk scores in women with different PCOS phenotypes.

Characteristics	Phenotype A (n=104)	Phenotype B (n=36)	Phenotype C (n=19)	Phenotype D (n=47)	P
Age (years)	28.05 ± 4.81	29.19 ± 6.23	28.00 ± 3.42	28.34 ± 5.73	0.711
BMI (kg/m^2^)	27.68 ± 5.84	27.43 ± 5.17	26.23 ± 4.68	22.78 ± 4.75	<0.001
WC (cm)	84.34 ± 13.97	86.41 ± 13	81.65 ± 10.21	73.75 ± 11.56	<0.001
SBP (mmHg)	107.59 ± 16.03	109.68 ± 14.35	109.71 ± 11.15	99.03 ± 10.13	0.001
DBP (mmHg)	71.6 ± 11.79	72.24 ± 11.73	73.29 ± 10.39	65.6 ± 9.13	0.008
UA (umol/L)	360.65 ± 85.92	367.76 ± 78.15	322.16 ± 75.77	289.87 ± 62.4	<0.001
TG (mmol/L)	1.24(0.71,1.77)	1.23(0.87,2.04)	1.19(0.68,1.54)	0.82(0.57,1.34)	0.006
TC (mmol/L)	4.82 ± 0.70	5.08 ± 1.02	4.75 ± 0.76	4.69 ± 1.00	0.213
HDL-C (mmol/L)	1.43 ± 0.42	1.33 ± 0.38	1.51 ± 0.38	1.69 ± 0.49	<0.001
LDL-C (mmol/L)	2.90 ± 0.62	3.22 ± 0.95	2.77 ± 0.59	2.63 ± 0.83	0.005
FBG (mmol/L)	5.01 ± 0.59	5.22 ± 0.95	4.73 ± 0.42	4.80 ± 0.62	0.011
2-h BG (mmol/L)	7.04 ± 2.35	8.26 ± 4.23	6.12 ± 1.41	5.71 ± 1.62	<0.001
FINS (uIU/mL)	15.19(10.64,30.19)	22.62(14.87,29.48)	14.56(10.44,17.83)	8.06(6.40,12.45)	<0.001
2-h INS (uIU/mL)	110.30(60.65, 202.50)	121.30(83.95, 253.90)	94.45(52.81, 169.50)	50.10(37.72, 79.54)	<0.001
HbA1C (%)	5.38 ± 0.47	5.39 ± 0.57	5.18 ± 0.30	5.21 ± 0.32	0.068
HOMA-IR	3.43(2.32,6.97)	4.90(3.01,6.74)	3.01(2.23,3.38)	1.71(1.30,2.58)	<0.001
ISI-Matsuda	3.23(1.51,5.23)	2.35(1.47,3.99)	3.71(2.49,6.79)	7.30(5.87,8.76)	<0.001
LH/FSH	1.97(1.38,2.99)	1.44(0.76,2.14)	1.28(1.01,2.20)	1.71(1.09,2.83)	0.048
TT (ng/dL)	69.03(51.67,84.90)	55.06(41.05,78.55)	59.73(38.76,95.67)	50.18(34.61,64.99)	<0.001
AMH (ng/ml)	7.90(5.68,11.91)	7.31(3.42,9.60)	8.23(6.90,12.48)	9.14(5.25,13.82)	0.270
10-year CVD risk score (%)	0.10(0,0.40)	0.10(0.10,0.88)	0.20(0.10,0.30)	0.10(0,0.10)	0.009
Lifetime CVD risk score (%)	15.55(10.00,24.10)	17.65(10.18,27.08)	17.30(12.60,22.13)	9.90(7.70,13.90)	<0.001

Normally distributed continuous variables are presented as mean ± SD, and non-normally distributed variables are presented as median (25th percentile, 75th percentile). 200 for UA, 198 for HDL-C, LDL-C, 10-year CVD risk score, and Lifetime CVD risk score. P<0.05 were considered significant.

**Figure 3:**
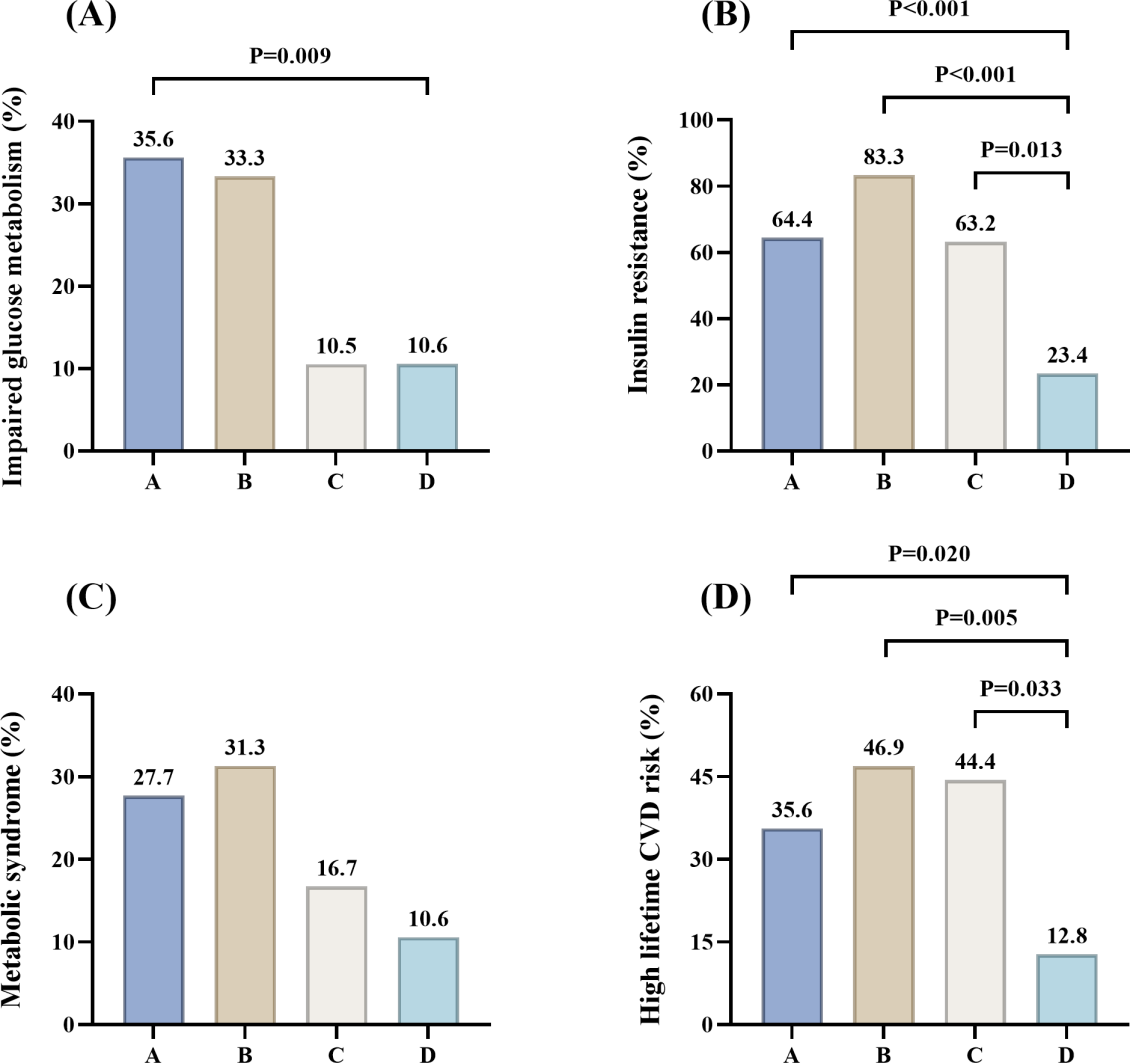
Proportions of women with impaired glucose metabolism, insulin resistance, metabolic syndrome and high lifetime CVD risk across different PCOS phenotypes. **(A)** Impaired glucose metabolism; **(B)** Insulin resistance; **(C)** Metabolic syndrome, with 198 patients, including 101, 32, 18, and 47 in phenotypes (A–D); **(D)** High lifetime CVD risk, with 198 patients, including 101, 32, 18, and 47 in phenotypes (A–D). P< 0.05 was considered significant.

FBG levels were significantly higher in phenotypes B than C and D (P = 0.009 and P = 0.004). 2-h BG levels were significantly higher in phenotype B than in A, C, and D (P = 0.024, P = 0.008, and P< 0.001). Phenotype A also had significantly higher 2-h BG levels than D (P = 0.010). The prevalence of impaired glucose metabolism was highest in phenotypes A (37, 35.6%), followed by B (12, 33.3%),D (5, 10.6%) and C (2, 10.5%) (A vs D, P = 0.009) (**Figure 3A**). FINS and 2-h INS levels were significantly higher in phenotypes A, B, and C compared wiht D (FINS: P<0.001, P<0.001, P = 0.033; 2-h INS: all P<0.001). HOMA-IR was significantly higher, while ISI-Matsuda was significantly lower in phenotypes A and B compared to D (all P< 0.001). The prevalence of IR was higher in phenotypes B (30, 83.3%), A (67, 64.4%) and C (12, 63.2%) compared with D (11, 23.4%) (P<0.001, P<0.001 and P = 0.013) (**Figure 3B**). The prevalence of MetS was highest in phenotypes B (10, 31.3%), followed by A (28, 27.7%), C (3, 16.7%), and D (5, 10.6%), with no statistically significant differences observed (**Figure 3C**).

TT levels were significantly higher in phenotype A compared with D (P<0.001). Although an overall difference in the LH/FSH ratio was observed (P = 0.048), with phenotypes A and D showing higher values than B and C, *post-hoc* analyses did not show significant differences. No significant differences were found in age, TC, HbA1c, or AMH levels.

### 3.2 CVD risk assessment in women with different PCOS phenotypes

As shown in **Table 1**, 10-year CVD risk scores were significantly higher in phenotypes A, B, and C compared with D (P = 0.046, P = 0.049, and P = 0.037). Similarly, lifetime CVD risk scores were significantly higher in phenotypes A, B, and C compared with D (P< 0.001, P = 0.001, and P = 0.007), with median values of 15.55%, 17.65%, 17.30%, and 9.90%.

All participants had low 10-year CVD risk. However, the proportion of high lifetime CVD risk was higher in phenotypes B (15, 46.9%), C (8, 44.4%), and A (36, 35.6%) compared with D (6, 12.8%) (P = 0.020, P = 0.005, and P = 0.033) (**Figure 3D**).

Multiple clinical parameters showed significant positive correlations with lifetime CVD risk score, including BMI (r=0.794, P<0.001), UA (r=0.505, P<0.001), TG (r=0.550, P<0.001), LDL-C (r=0.289, P<0.001), FBG (r=0.410, P<0.001), 2-h BG (r=0.503, P<0.001), FINS (r=0.707, P<0.001), 2-h INS (r =0.509, P<0.001), HbA1c (r=0.387, P<0.001), and HOMA-IR (r=0.713, P<0.001). In contrast, inverse correlations were observed for LH/FSH ratio (r=-0.263, P<0.001), AMH (r=-0.203, P = 0.005), and ISI-Matsuda (r=-0.685, P<0.001). No significant association was found between TT and lifetime CVD risk score.

### 3.3 Association between PCOS phenotypes and CVD Risk

We examined the association between PCOS phenotypes and high lifetime CVD risk, adjusting for diet, physical activity, and medication use as potential confounders. Firth logistic regression was used to estimate the ORs for high lifetime CVD risk across phenotype groups (**Table 2**). In the unadjusted Model 1, women with phenotypes A, B, and C had significantly higher ORs for high lifetime CVD risk compared with D (P = 0.003, P = 0.001 and P = 0.008). After adjusting for diet and physical activity, phenotypes A, B, and C remained significantly associated with higher ORs for high lifetime CVD risk compared with D (P = 0.005, P = 0.006, and P = 0.009). Further adjustment for medication use did not alter these associations: phenotype A (OR 3.18, 95% CI: 1.30-8.84, P = 0.010), phenotype B (OR 4.90, 95% CI: 1.58-16.44, P = 0.006), and phenotype C (OR 4.67, 95% CI: 1.39-16.64, P = 0.013).

**Table 2:** Firth logistic regression of the association between PCOS phenotypes and high lifetime CVD risk.

Models	Phenotype D (n=47)	Phenotype A (n=101)	Phenotype B (n=32)	Phenotype C (n=18)
High lifetime CVD risk	6(12.8)	36(35.6)	15(46.9)	8(44.4)
Model 1	1.00	3.66 (1.54-9.93)^**^	5.65 (2.01-17.56)^**^	5.17 (1.54-18.40)^**^
Model 2	1.00	3.45 (1.43-9.53)^**^	4.94 (1.59-16.53)^**^	4.95 (1.48-17.56)^**^
Model 3	1.00	3.18 (1.30-8.84)^*^	4.90 (1.58-16.44)^**^	4.67 (1.39-16.64)^*^

Data are presented as n (%) or OR (95% CI). Model 1: unadjusted; Model 2: adjusted for diet and physical activity; Model 3: adjusted for diet, physical activity, and medication use (antihypertensives and lipid-lowering drugs). A total of 198 patients, with 101, 32, 18, and 47 in phenotypes A, B, C, and D. *P<0.05, **P<0.01.

We further conducted mediation analysis to explore the relationship between metabolic factors, PCOS phenotypes, and high lifetime CVD risk. Significant mediating effects were observed for BMI, HOMA-IR, and UA (**Table 3**). The indirect effect of BMI was significant for phenotypes A, B, and C compared with D: A (0.21, 95% CI: 0.11-0.29, P<0.001), B (0.26, 95% CI: 0.10-0.42, P<0.001), and C (0.14, 95% CI: 0.03-0.31, P = 0.018), with proportions mediated of 94.7%, 70.9%, and 42.9%. The direct effects of BMI were not significant across all phenotypes. The indirect effect of HOMA-IR was significant for phenotypes A and B compared with D: A (0.13, 95% CI: 0.05-0.20, P<0.001), B (0.18, 95% CI: 0.06-0.34, P<0.001), with proportions mediated of 56.2% and 65.1%. The indirect effect of HOMA-IR was not significant for phenotype C, but its direct effect was significant (P = 0.046), while the direct effects of HOMA-IR were not significant for phenotypes A and B. The indirect effect of UA was significant for phenotypes A and B compared with D: A (0.13, 95% CI: 0.07-0.20, P<0.001), B (0.16, 95% CI: 0.05-0.31, P = 0.004), with proportions mediated of 59.8% and 46.3%. The indirect effect of UA for phenotype C was not significant, but its direct effect was significant (P = 0.042), while the direct effects of UA were not significant for phenotypes A and B.

**Table 3:** Mediation effects of BMI, HOMA-IR and UA in the relationship between phenotypes and high lifetime CVD risk.

Mediating factors	Indirect effect	Direct effect	Total effect	Proportion mediated (%)
BMI				
Phenotype A	0.21 (0.11-0.29)^***^	0.01 (-1.34-0.18)	0.22 (0.07-0.36)^**^	94.7
Phenotype B	0.26 (0.10-0.42)^***^	0.11 (-0.10-0.31)	0.37 (0.14-0.56)^***^	70.9
Phenotype C	0.14 (0.03-0.31)^*^	0.19 (-0.07-0.43)	0.33 (0.06-0.57)^**^	42.9
HOMA-IR				
Phenotype A	0.13 (0.05-0.20)^***^	0.10 (-0.05-0.26)	0.23 (0.08-0.37)^**^	56.2
Phenotype B	0.18 (0.06-0.34)^***^	0.10 (-0.12-0.34)	0.28 (0.09-0.51)^*^	65.1
Phenotype C	0.05 (-0.05-0.21)	0.30 (0.00-0.47)^*^	0.35 (0.03-0.55)^*^	14.2
UA				
Phenotype A	0.13 (0.07-0.20)^***^	0.09 (-0.06-0.24)	0.22 (0.09-0.36)^**^	59.8
Phenotype B	0.16 (0.05-0.31)^**^	0.19 (-0.05-0.43)	0.35 (0.13-0.56)^**^	46.3
Phenotype C	0.06 (-0.01-0.18)	0.26 (0.01-0.52)^*^	0.32 (0.09-0.58)^*^	19.5

The indirect, direct, and total effects from the mediation analysis comparing phenotypes A, B, and C with phenotype D are presented as estimates with 95% CI. The proportion mediated represents the percentage of the total effect mediated by each variable. *P< 0.05, **P< 0.01, ***P< 0.001.

## 4 Discussion

To our knowledge, this cross-sectional study is the first to demonstrate significant differences in predicted CVD risk among Chinese women with different PCOS phenotypes. Both 10-year and lifetime CVD risk scores were significantly higher in phenotypes A, B, and C compared with D. Furthermore, women with phenotypes A, B, and C had a markedly higher prevalence of high lifetime CVD risk than those with D. These findings are consistent with the more pronounced clinical abnormalities observed in phenotypes A, B, and C compared with D.

In this study, phenotype A was the most prevalent, followed by D, B, and C. A meta-analysis showed that phenotype A is consistently the most common across diverse populations (29). Although the prevalence of phenotypes B, C, and D varies, our findings align with those of Bizoń et al. (30). These differences may be due to whether women were assessed during clinical consultations or routine health evaluations (31). Moreover, our study demonstrated a higher degree of obesity in women with phenotypes A, B, and C compared to D, with phenotype B being particularly severe. This is consistent with the findings of Jamil et al. (32) and Gupta et al. (33), which showed that women with hyperandrogenic phenotypes have higher BMI, WC, and the prevalence of obesity. Additionally, women with phenotypes A, B, and C had elevated blood pressure compared to D, a finding supported by several studies. For instance, studies from Iran, India, and Iraq reported higher SBP and DBP in women with phenotypes A and B (34–36).

Our results indicated more severe hyperuricemia, dyslipidemia and dysglycemia in women with PCOS phenotypes A and B compared to D, with B being particularly severe, and C showing intermediate levels. Liangshan et al. (37) reported a positive association between hyperandrogenism, UA levels, and hyperuricemia prevalence in women with PCOS. Similarly, Ma et al. (38) and Tripathy et al. (35) reported that phenotypes A and B are associated with higher prevalence of dyslipidemia than other phenotypes. Persson et al. (39) identified an increased risk of type 2 diabetes in women with hyperandrogenic phenotypes compared to non-androgenic phenotypes. Furthermore, a similar pattern was observed for IR, with phenotypes A, B, and C showing more severe IR and reduced insulin sensitivity than D, particularly in phenotype B. Although some studies found no significant intergroup differences in IR (40, 41), others reported significantly higher IR prevalence in phenotypes A and B compared to D (42–44). Although the prevalence of MetS showed no significant difference, it was higher in phenotypes A and B, with phenotype B being the most severe, indicating more severe metabolic abnormalities in A and B, consistent with previous studies (34, 45). Additionally, despite higher TT levels in phenotypes A, B, and C compared with D, a significant difference was only observed between A and D. This is consistent with previous studies, which found that phenotype A had the highest TT levels (46), with elevated TT levels also observed in phenotypes A, B, and C compared with D (40). Phenotypes A and D also exhibited numerically higher LH/FSH ratios than B and C, a pattern in line with previous research (32, 47).

The finding that women with PCOS phenotypes A, B, and C have higher 10-year and lifetime CVD risk scores, with more classified as high lifetime CVD risk compared with D, particularly phenotype B, aligns with the metabolic characteristics of these subgroups. Most studies have reported that hyperandrogenic phenotypes A and B are associated with higher metabolic risk, while phenotype D has the lowest metabolic and CVD risk (32, 43). Krentowska et al. (29) further suggested that women with hyperandrogenic phenotypes (A, B, and C) are at higher risk of adverse metabolic outcomes. Pluta et al. (44) identified phenotype B as having the highest proportion of abnormal metabolic parameters, including elevated IR, FBG, and abnormal HDL levels, making it the most metabolically burdened subgroup with a significantly increased risk of cardiovascular complications. This is consistent with our findings, where phenotype B exhibits the most severe obesity and metabolic abnormalities, which likely contribute to its highest lifetime CVD risk scores and proportion of high lifetime CVD risk. Additionally, the relatively older age of phenotype B may further contribute to their elevated lifetime CVD risk.

Firth logistic regression results showed that after adjusting for diet, physical activity, and medication use, all hyperandrogenic phenotypes (A, B, and C) were significantly associated with higher ORs for high lifetime CVD risk compared to phenotype D, with phenotype B exhibiting the strongest association. This is consistent with previous findings, in which phenotypes A, B, and C exhibited more severe metabolic abnormalities, with phenotype B being the most severe, indicating a higher CVD risk. It also suggests that PCOS phenotypes may be independent risk factors for CVD, regardless of lifestyle and medication use. Several studies have shown the influence of lifestyle factors on cardiometabolic risk in women with PCOS. Evidence suggests that a healthy diet and regular physical activity improve IR, body composition, and other cardiometabolic risk markers in this population (48). Structured exercise interventions improve cardiorespiratory fitness, potentially reducing cardiometabolic burden (49). However, our findings highlight the importance of phenotype in CVD risk, which may be influenced by the generally low physical activity levels and unhealthy diet in PCOS women.

Correlation analysis results suggested that various metabolic factors were associated with CVD risk in women with PCOS. We further explored the role of metabolic factors in the differences in CVD risk across phenotypes. Mediation analysis results indicated that BMI plays a significant mediating role in high lifetime CVD risk across phenotypes A, B, and C, with the direct effect of phenotype being relatively smaller. Similar findings were observed for HOMA-IR and UA, which also showed significant mediating effects in phenotypes A and B. Obesity is a major contributor to cardiometabolic abnormalities in women with PCOS. A meta-analysis found that increased BMI in PCOS women is associated with greater cardiometabolic abnormalities, suggesting BMI partly mediates the heightened cardiovascular risk (50), and while PCOS is associated with CVD risk even after adjusting for BMI, higher BMI worsens these abnormalities, emphasizing its significant contribution to cardiovascular risk (51). IR is highly prevalent occurring in 60%-90% of women with PCOS (52), and several studies have demonstrated positive correlations between HOMA-IR and CVD risk factors (53). Furthermore, Elevated UA levels are common in PCOS and are associated with metabolic syndrome components, suggesting that UA may contribute to or reflect an increased cardiometabolic risk in PCOS women (54). The role of PCOS as an independent CVD risk factor remains debated. Berni et al. (55) and Glintborg et al. (56) reported that women with PCOS are at higher CVD risk even after adjusting for obesity and other factors, suggesting PCOS itself is an independent risk factor. In contrast, Pandurevic et al. (57) found that obesity, not phenotype, was the primary determinant of early CVD markers in PCOS. A Mendelian randomization study also found no association between genetically predicted PCOS and CVD risk, suggesting that PCOS may not directly increase risk. Instead, features of PCOS—particularly obesity—may explain the observed link to CVD (58). In our study, metabolic abnormalities varied across different PCOS phenotypes, consistent with their CVD risk. This highlights the central role of phenotype, with obesity and other metabolic factors mediating CVD risk in women with PCOS, emphasizing the importance of phenotype-specific management along with weight and metabolic control.

Although this study did not include non-PCOS populations for comparison, women with PCOS exhibited higher lifetime CVD risk scores compared to studies of healthy populations. A study of 1,688 women who participated in physical examinations found that the lifetime CVD risk scores for those under 35 was 8.1% ± 2.9%, and for those aged 35 to 44, it was 9.4% ± 4.3% (59).In our study, phenotypes B and C had CVD risk scores 1.8 to 2.2 times higher than those of the two comparison groups, while phenotype A had scores 1.7 to 1.9 times higher. Even phenotype D, with the lowest lifetime CVD risk score, had higher scores than both age groups. At present, few studies have investigated CVD risk prediction in Chinese women with different PCOS phenotypes. This study provides valuable insights by evaluating the significant differences in predicted CVD risk across phenotypic subgroups. The findings emphasize the need for phenotype-specific CVD risk assessment to better identify high-risk women for targeted interventions, supporting more personalized treatment and prevention, and highlighting the importance of weight and metabolic management, particularly in high-risk phenotypes.

Several limitations of this study should be acknowledged. First, the study population is relatively young, and due to the unique metabolic, endocrine, and reproductive traits of PCOS, current cardiovascular risk tools have not been validated for PCOS patients. We plan to expand the cohort and conduct long-term follow-up to further validate the model’s effectiveness. Secondly, this study did not include a control group, which may limit the interpretation and clinical applicability of the findings. Future research will include a matched control group to better assess the CVD risk differences between PCOS patients and non-PCOS individuals. Third, the modest sample size and imbalanced phenotype distribution may affect the results, necessitating further expansion to improve reliability. Finally, as a cross-sectional study, it limits understanding cause-and-effect relationships.

In summary, this study characterized the distinct clinical profiles and CVD risk predictions among Chinese women with different PCOS phenotypes. Women with phenotypes A, B, and C exhibited more pronounced clinical abnormalities and significantly higher CVD risk than those with phenotype D. PCOS phenotypes were significantly associated with high lifetime CVD risk, with BMI, HOMA-IR, and UA play a significantly mediating role between phenotypes and CVD risk. Therefore, CVD risk across PCOS phenotypes should be regularly assessed to guide individualized treatment strategies, with an emphasis on weight and metabolic management.

## Data Availability

The raw data supporting the conclusions of this article will be made available by the authors, without undue reservation.
